# Supplementary material for: In situ structure and assembly of the multidrug efflux pump AcrAB-TolC
Source: Nat Commun. 2019 Jun 14;10:2635. doi: 10.1038/s41467-019-10512-6 (PMC6570770; doi:10.1038/s41467-019-10512-6)
Supplement: Supplementary file 1 — Supplementary Information [file 41467_2019_10512_MOESM1_ESM.pdf]

Supplementary Information for

Shi *et al.*'s manuscript entitled "In situ structure and assembly of multidrug efflux pump AcrAB-TolC"

Supplementary Figures and Tables

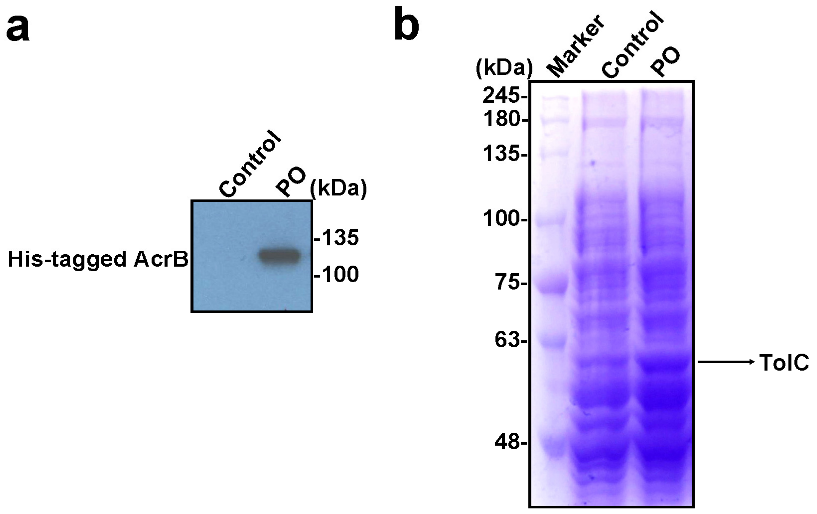

Supplementary Figure 1 | Overexpression of AcrA, AcrB, and TolC in BL21(DE3) cells. Expression plasmids pAcBH encoding AcrA and AcrB and pRSF-*tolC* were cotransformed into BL21 (DE3) cells. Protein expression was induced with 0.1 mM IPTG at 20 °C overnight. BL21 (DE3) cells without plasmid transformation were used as the control. (**a**) Immunoblotting results of AcrB overexpression by using anti-his antibodies. Source data are provided as a Source Data file. (**b**) SDS-PAGE analysis result of TolC overexpression. PO: pump overexpression.

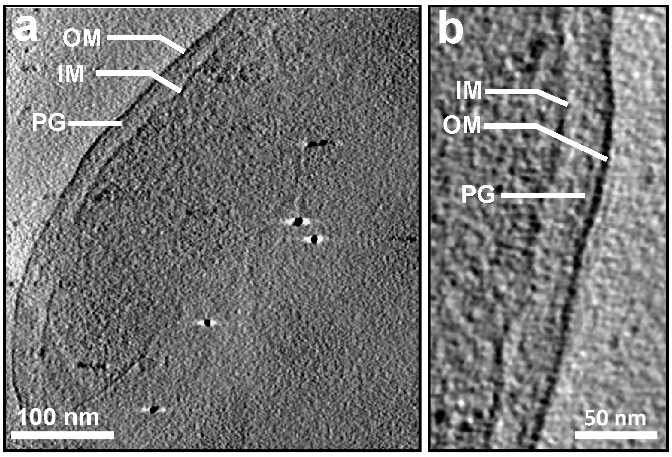

Supplementary Figure 2 | Visualizing the cell envelope of *E. coli* BL21 (DE3) wild type cells. (**a**) A single slice from a tomogram of *E. coli*. (**b**) Zoomed in side view of the cell envelope. OM, outer membrane; IM, inner membrane; PG, peptidoglycan.

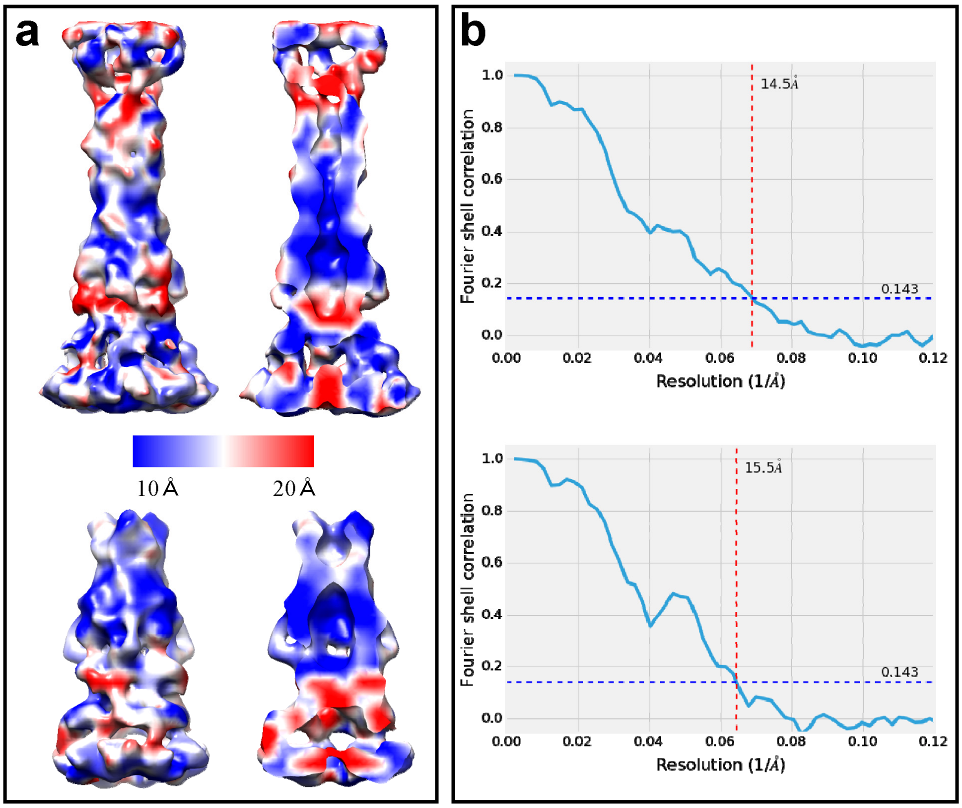

Supplementary Figure 3 | Local and global resolution of the subtomogram average. (a) Local resolution of the averaged structure overlaid on the isosurface rendering. Upper: full pump; lower: AcrAB subcomplex. (**b**) Global gold-standard FSC curve of the averaged structure under a soft mask. Upper: full pump; lower: AcrAB subcomplex.

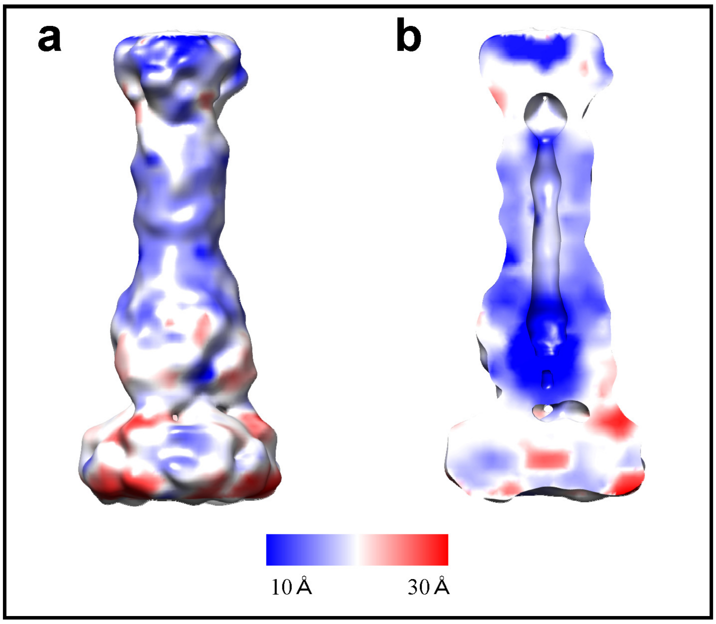

Supplementary Figure 4 | Local resolution of the subtomogram average of the fully assembled pump under inhibitor treatment. (**a**) Local resolution of the averaged structure overlaid on the isosurface rendering. (**b**) A slice through the density map of a.

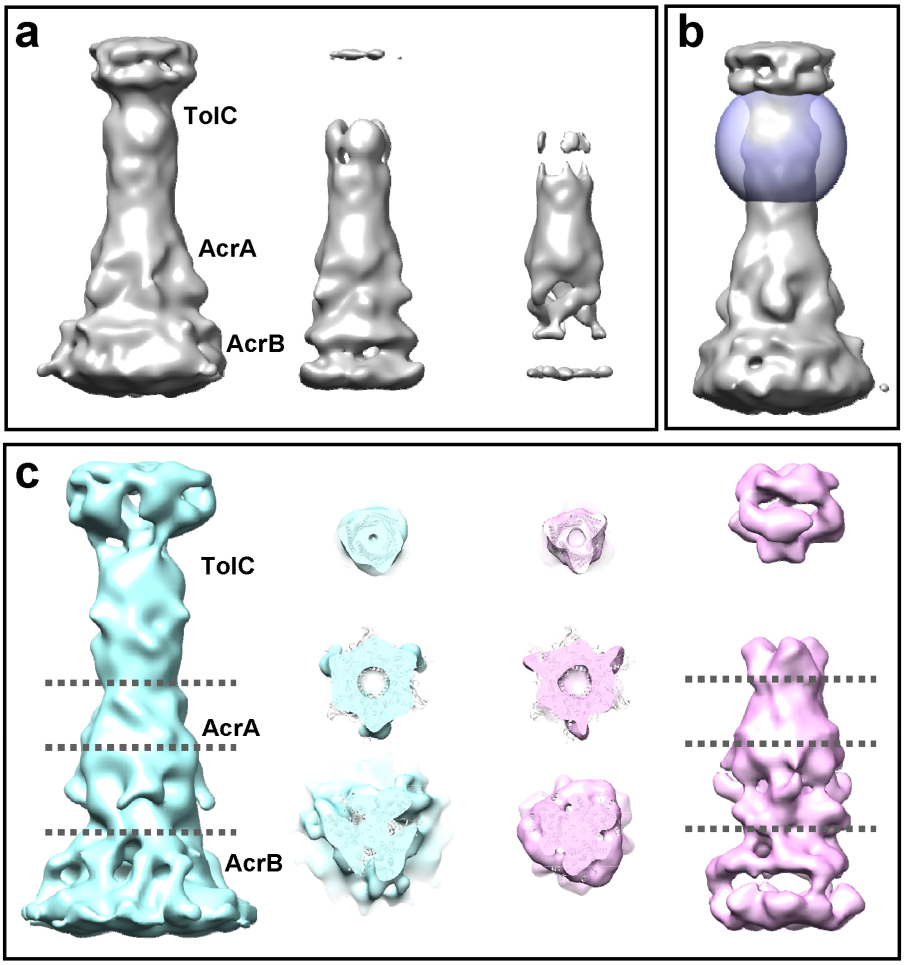

Supplementary Figure 5 | Focused classification to evaluate particle composition. (**a**) Reconstructions for the pump at different isosurface level (from left to right, with isosurface threshold increasing). (**b**) Mask for focused classification (blue) overlaid on the structure. (**c**) Cross section views of the averaged density maps from the two classes at the AcrA and AcrB subunit, fitted with the PDB model (5V5S). Cyan: fully assembled pump; pink: AcrAB subcomplex.

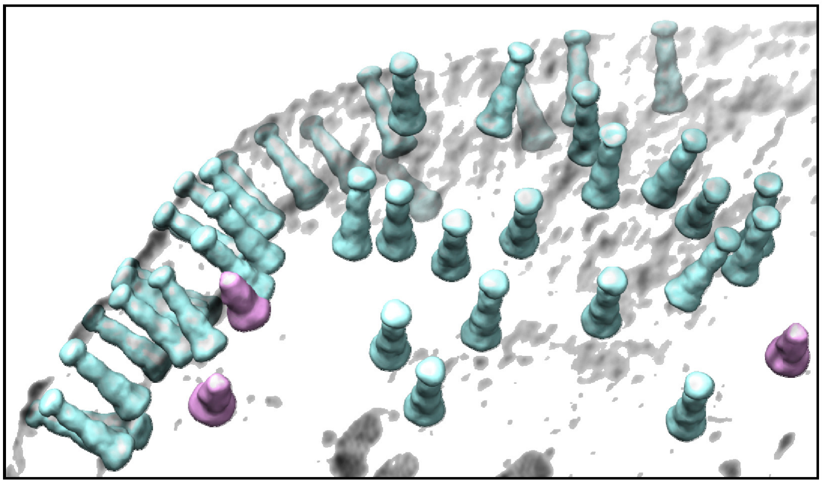

Supplementary Figure 6 | Segmentation showing fully assembled AcrAB-TolC pump and AcrAB subcomplex distributed in their original cell.

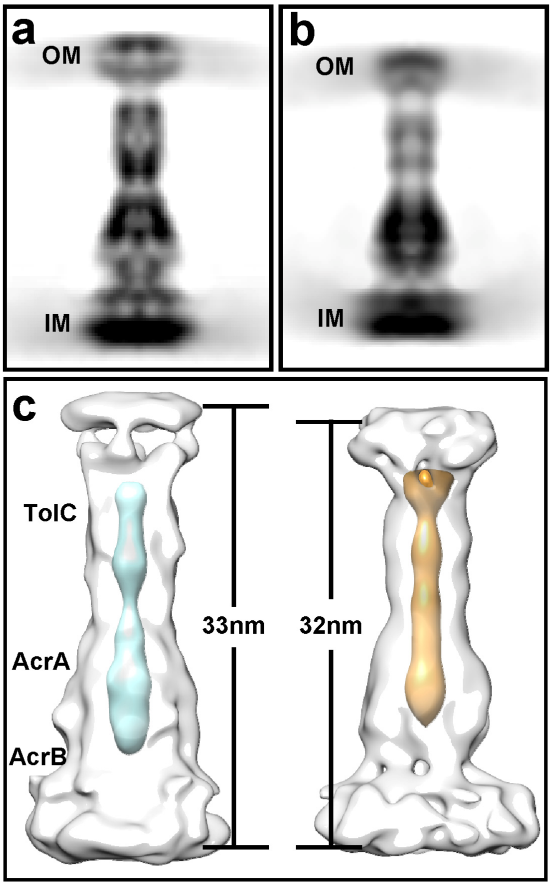

Supplementary Figure 7 | Comparison of the structure solved with antibiotics (closed state) treatment and with AcrB inhibitor (open state). (**a**) Projection of the full pump structure at closed state. (**b**) Projection of the full pump structure at open state. (**c**) Three-dimensional density maps of the closed (left) and open (right) state pump, both filtered to 21 Å, with the colored inner channel overlaid.

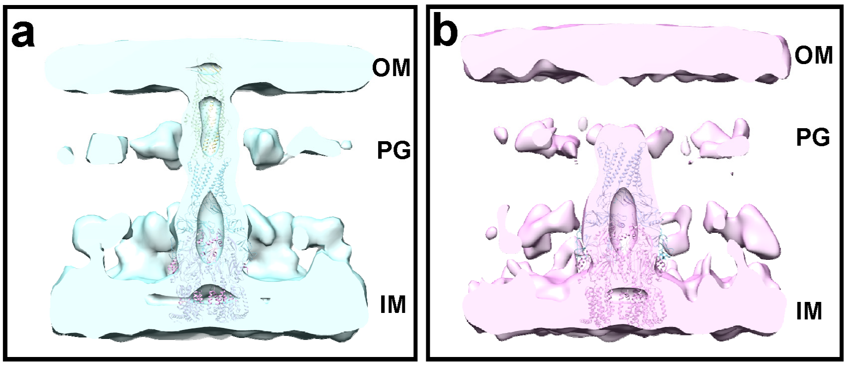

Supplementary Figure 8 | Reconstructions of AcrAB-TolC efflux pump in their surrounding environment. Density maps of the two classes refined without a mask fitted with the PDB model (5V5S), show the location of the peptidoglycan layer. (**a**) Fully assembled pump (cyan). (**b**) AcrAB subcomplex (pink).

a

3::sp|P02930|TOLC_ECOLI **Mass:** 53708 **Score:** 1389 **Matches:** 35(32) **Sequences:** 25(24) **emPAI:** 11.12
Outer membrane protein TolC OS=Escherichia coli (strain K12) OX=83333 GN=tolC PE=1 SV=3
Check to include this hit in error tolerant search or archive report

Query	Observed	Mr(expt)	Mr(calc)	ppm	Miss	Score	Expect	Rank	Unique	Peptide
1653	559.3140	1116.6135	1116.6139	-0.33	0	54	6.2e-05	1	U	Y.KQAVVSAQSSL.D
1728	566.7918	1131.5691	1131.5706	-1.34	0	(25)	0.092	1	U	L.KEAEKRNL.S + DTSSP_Cross_link_Carbamidomethyl (K)
1861	578.8193	1155.6241	1155.6247	-0.53	0	46	0.00032	1	U	F.EKINEARSPL.L
1886	580.3356	1158.6566	1158.6609	-3.69	0	24	0.043	1	U	Y.SVGTRTIVDVL.D
2230	605.8430	1209.6715	1209.6717	-0.19	0	48	0.0001	1	U	F.KTDKPQPVNAL.L
2486	415.8922	1244.6547	1244.6546	0.05	1	26	0.053	1	U	L.LKEAEKRNL.S + DTSSP_Cross_link_Carbamidomethyl (K)
2687	639.2931	1276.5717	1276.5718	-0.01	0	(32)	0.022	1	U	Y.DDSNMGQNKVGL.S
2688	639.3015	1276.5883	1276.5903	-1.55	0	34	0.015	1	U	L.KEAEKRNL.S + 2 DTSSP_Cross_link_Carbamidomethyl (K)
2692	639.3332	1276.6518	1276.6523	-0.40	1	47	0.00065	1	U	Y.NAKQELANARY.N
2822	647.2885	1292.5625	1292.5667	-3.26	0	50	0.00023	1	U	Y.DDSNMGQNKVGL.S + Oxidation (M)
2877	651.3222	1300.6298	1300.6299	-0.04	1	35	0.01	1	U	L.SYTQAQKEAIY.R
3035	661.8308	1321.6471	1321.6514	-3.26	0	22	0.31	1	U	L.QEKAAGIQDVTY.Q
3048	662.3846	1322.7547	1322.7558	-0.80	1	51	3.4e-05	1	U	F.KTDKPQPVNALL.K
3435	690.8448	1379.6751	1379.6681	5.14	0	43	0.0025	1	U	F.NNINASISSINAY.K
3862	724.8793	1447.7440	1447.7419	1.42	0	92	2.5e-08	1	U	L.VAITDVQNARAQY.D
3972	734.8949	1467.7752	1467.7755	-0.19	1	(37)	0.0035	1	U	F.KTDKPQPVNALL.K + DTSSP_Cross_link_Carbamidomethyl (K)
3975	735.3501	1468.6856	1468.6868	-0.76	2	32	0.036	1	U	F.SSLSQAENLMQVY.Q
4112	746.3549	1490.6953	1490.6961	-0.53	0	79	6.7e-07	1	U	Y.RDANGINSNATSASL.Q + Deamidated (NQ)
4113	746.3575	1490.7004	1490.6961	2.91	0	(37)	0.011	1	U	Y.RDANGINSNATSASL.Q + Deamidated (NQ)
4341	768.8980	1535.7813	1535.7831	-1.16	1	78	6.7e-07	1	U	L.TLQEKAAGIQDVTY.Q
4342	769.3738	1536.7330	1536.7355	-1.60	0	(59)	7.4e-05	1	U	Y.QGGMVNSQVKQAQY.N
4418	777.3720	1552.7295	1552.7304	-0.60	0	62	4.6e-05	1	U	Y.QGGMVNSQVKQAQY.N + Oxidation (M)
4693	804.9236	1607.8327	1607.8307	1.26	2	40	0.0036	1	U	L.RQITGNYYPELAAL.N
5577	881.4841	1760.9536	1760.9520	0.89	1	72	1.1e-06	1	U	Y.SVGTRTIVDVLDATTTL.Y
5842	916.4874	1830.9603	1830.9588	0.82	1	127	6e-12	1	U	F.NVGLVAITDVQNARAQY.D
5843	611.3275	1830.9607	1830.9588	1.04	1	(62)	1.9e-05	1	U	F.NVGLVAITDVQNARAQY.D
5919	927.9475	1853.8805	1853.8829	-1.31	1	91	6.6e-08	1	U	Y.KQAVVSAQSSLDAMEAGY.S
5979	935.9656	1869.9167	1869.9180	-0.70	1	66	1.6e-05	1	U	L.ANEVTARNNLDNAVEQL.R
6138	963.0156	1924.0166	1924.0153	0.63	2	57	5e-05	1	U	Y.SVGTRTIVDVLDATTTLY.N
6364	1000.4556	1998.8966	1998.9026	-3.03	1	(71)	6.4e-06	1	U	Y.KQAVVSAQSSLDAMEAGY.S + DTSSP_Cross_link_Carbamidomethyl (K)
7088	1150.0791	2298.1436	2298.1451	-0.65	2	67	1.4e-05	1	U	Y.DTVLANEVTARNNLDNAVEQL.R
7093	767.3895	2299.1468	2299.1292	7.66	2	(23)	0.33	1	U	Y.DTVLANEVTARNNLDNAVEQL.R + Deamidated (NQ)
7456	636.3040	2541.1867	2541.1878	-0.41	1	(36)	0.028	1	U	Y.SGSKTRGAAGTQYDDSNMGQNKVGL.S
7471	640.3030	2557.1831	2557.1827	0.14	1	56	0.00029	1	U	Y.SGSKTRGAAGTQYDDSNMGQNKVGL.S + Oxidation (M)
7472	853.4025	2557.1856	2557.1827	1.13	1	(41)	0.0084	1	U	Y.SGSKTRGAAGTQYDDSNMGQNKVGL.S + Oxidation (M)

b

4::sp|P0AE06|ACRA_ECOLI **Mass:** 42301 **Score:** 767 **Matches:** 32(29) **Sequences:** 16(14) **emPAI:** 10.09
Multidrug efflux pump subunit AcrA OS=Escherichia coli (strain K12) OX=83333 GN=acrA PE=1 SV=1
Check to include this hit in error tolerant search or archive report

Query	Observed	Mr(expt)	Mr(calc)	ppm	Miss	Score	Expect	Rank	Unique	Peptide
857	487.2687	972.5229	972.5240	-1.09	1	26	0.041	1	U	L.KQELANGTL.K
1640	557.8320	1113.6494	1113.6506	-1.09	0	49	9.6e-05	1	U	L.KAGDRVVISGL.Q
2456	621.8619	1241.7092	1241.7092	0.05	2	(41)	0.00069	1	U	L.RLKQELANGTL.K
2467	622.3532	1242.6919	1242.6932	-1.04	2	(47)	0.00023	1	U	L.RLKQELANGTL.K + Deamidated (NQ)
3347	684.3378	1366.6611	1366.6616	-0.36	1	67	9.7e-06	1	U	F.KEGSDIEAGVSLY.Q
3477	694.3727	1386.7308	1386.7289	1.38	2	50	0.00029	1	U	L.RLKQELANGTL.K + DTSSP_Cross_link_Carbamidomethyl (K)
4120	746.3862	1490.7579	1490.7617	-2.54	0	40	0.0043	1	U	L.ITSDGIKFPQDGTL.E
4407	775.9718	1549.9290	1549.9304	-0.87	0	31	0.00079	1	U	Y.RIAEVRPQVSGIIL.K
4409	517.6504	1549.9293	1549.9304	-0.68	0	(22)	0.0065	1	U	Y.RIAEVRPQVSGIIL.K
5606	884.4441	1766.8736	1766.8727	0.53	1	33	0.03	1	U	L.ITSDGIKFPQDGTLEF.S
5664	893.4141	1784.8136	1784.8139	-0.18	1	24	0.32	1	U	L.EFSDVTVDQTTGCITL.R
5818	609.3182	1824.9327	1824.9345	-0.98	1	(42)	0.0026	1	U	L.RAIFPNPDHTLLPGMF.V
5819	913.4756	1824.9366	1824.9345	1.16	1	(36)	0.011	1	U	L.RAIFPNPDHTLLPGMF.V
5881	614.6508	1840.9305	1840.9294	0.56	1	48	0.00077	1	U	L.RAIFPNPDHTLLPGMF.V + Oxidation (M)
5882	921.4742	1840.9339	1840.9294	2.44	1	(40)	0.0041	1	U	L.RAIFPNPDHTLLPGMF.V + Oxidation (M)
5924	928.4946	1854.9747	1854.9799	-2.79	1	78	3.5e-07	1	U	Y.DSAKGDLAKAQAAANIAQL.T
5925	619.3334	1854.9785	1854.9799	-0.75	1	(63)	1.1e-05	1	U	Y.DSAKGDLAKAQAAANIAQL.T
6373	1001.0056	1999.9967	1999.9996	-1.47	1	(57)	0.0001	1	U	Y.DSAKGDLAKAQAAANIAQL.T + DTSSP_Cross_link_Carbamidomethyl (K)
7348	818.7704	2453.2893	2453.2914	-0.86	0	46	0.00067	1	U	L.VVGADDKVETRPIVASQAIGDKW.L
7521	867.1120	2598.3142	2598.3112	1.15	0	(45)	0.0016	1	U	L.VVGADDKVETRPIVASQAIGDKW.L + DTSSP_Cross_link_Carbamidomethyl (K)
7625	892.1520	2673.4341	2673.4337	0.14	1	67	3.5e-06	1	U	L.KQENGKAKVSLITSDGIKFPQDGTL.E
7626	669.3658	2673.4343	2673.4337	0.20	1	(46)	0.00046	1	U	L.KQENGKAKVSLITSDGIKFPQDGTL.E
7627	535.6952	2673.4396	2673.4337	2.18	1	(28)	0.027	1	U	L.KQENGKAKVSLITSDGIKFPQDGTL.E
7770	932.4926	2794.4558	2794.4573	-0.52	1	53	0.00015	1	U	Y.DQALADAQQANAAVTAAKAAVETARINL.A
7883	981.4824	2941.4253	2941.4274	-0.73	0	70	1.1e-05	1	U	L.TGCDDKQAQQGGQQMPAVGVVTVKTEPL.Q
8060	1048.5520	3142.6342	3142.6292	1.58	1	20	0.3	1	U	Y.TKVTSPISGRIGKSNVTEGALVQNGQATAL.A + Deamidated (NQ); DTSSP_Cross_link_Carbamidomethyl (K)
8224	696.5585	3477.7563	3477.7560	0.07	0	(33)	0.028	1	U	L.QKVRPGVQVKAQEVTADNNQQAASGAQPEQSKS.-
8225	870.4464	3477.7565	3477.7560	0.15	0	68	9.8e-06	1	U	L.QKVRPGVQVKAQEVTADNNQQAASGAQPEQSKS.-
8227	696.7571	3478.7493	3478.7400	2.67	0	(39)	0.0079	1	U	L.QKVRPGVQVKAQEVTADNNQQAASGAQPEQSKS.- + Deamidated (NQ)
8262	906.7007	3622.7736	3622.7758	-0.59	0	(33)	0.042	1	U	L.QKVRPGVQVKAQEVTADNNQQAASGAQPEQSKS.- + DTSSP_Cross_link_Carbamidomethyl (K)
8263	906.7020	3622.7789	3622.7758	0.87	0	(27)	0.18	1	U	L.QKVRPGVQVKAQEVTADNNQQAASGAQPEQSKS.- + DTSSP_Cross_link_Carbamidomethyl (K)
8265	907.2026	3624.7815	3624.7438	10.4	0	(48)	0.0013	1	U	L.QKVRPGVQVKAQEVTADNNQQAASGAQPEQSKS.- + 2 Deamidated (NQ); DTSSP_Cross_link_Carbamidomethyl (K)

Supplementary Figure 9 | Crosslinking of AcrA and TolC with PG in vivo as detected by LC/MS-MS analysis. (**a**) The MASCOT output for peptides from TolC for the SDS-PAGE extracted sample. (**b**) The MASCOT output for peptides from AcrA for SDS-PAGE extracted sample.

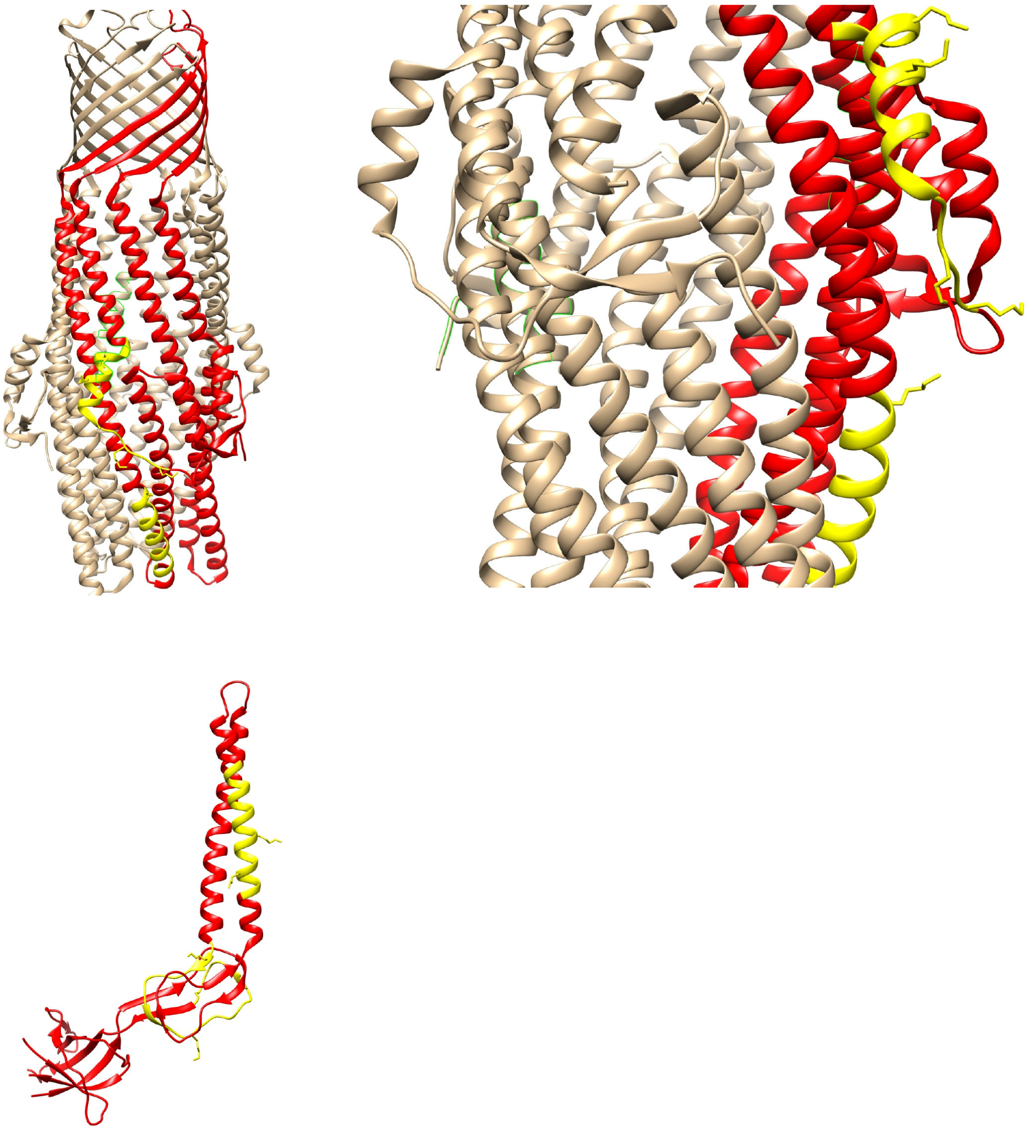

Supplementary Figure 10 | Mapping the binding sites of both AcrA and TolC with PG. The crystal structures of TolC trimer (upper panel, PDB: 1EK9) and AcrA (lower panel, PDB: 2F1M) with mapped peptides, highlighted in yellow. The candidate lysine residues that interact with the DTSSP bifunctional crosslinker are shown with side chains (K202, K205, K214, K218, K345 for TolC; K109, K114 in AcrA helical hairpin and K175, K186 in AcrA lipoyl domain). The right panels show zoomed views. For clarity, the mapped peptides are shown for only one protomer of the TolC trimer. The figures were prepared with UCSF Chimera.

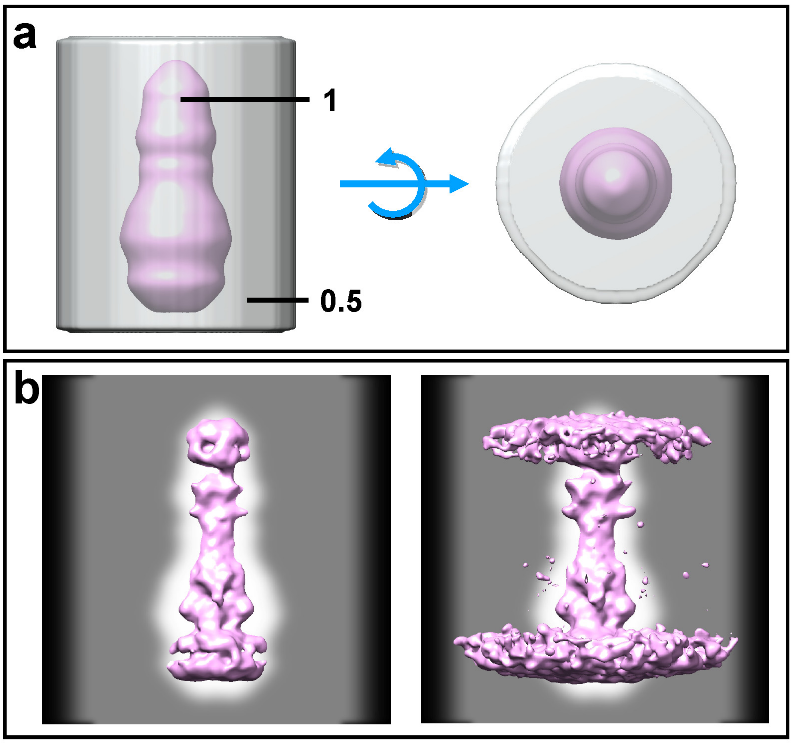

Supplementary Figure 11 | Masking strategy. (a) Top and side view of the two-level mask used for refinement. The pink density in the middle has the value of 1 and the grey density around has the value of 0.5. A soft falloff was applied at the interface between the pink and grey density, and at the edge of the grey density. (**b**) Two-dimensional slice of the mask in the X-Z direction and the averaged map at high and low isosurface threshold.

Supplementary Table 1 | Puromycin susceptibility of *E. coli* BL21 (DE3) cells over-expressing AcrAB-TolC pump.

**Proteins expressed**	**MIC (μg/ml)**	
	In the absence of MBX3132	In the presence of MBX3132 (1.4 μg/ml)
-	32	8
AcrA, AcrB, TolC	64-128	8

Supplementary Table 2 | Cryo-ET data analysis and validation statistics.

	AcrAB-TolC open state (EMD-0533)	AcrAB-TolC close state (EMD-0532)	AcrAB subcomplex (EMD-0531)
**Data collection and processing**			
Magnification	12,000	10,000	10,000
Voltage (kV)	300	300	300
Electron exposure (e–/Å^2)	76	76	76
Defocus range (µm)	−3 to −6	−3 to −6	−3 to −6
Pixel size (Å)	2.75	3.366	3.366
Symmetry imposed	C3	C3	C3
Initial particle images (no.)	678	1,321	1,321
Final particle images (no.)	678	800	561
Map resolution (Å)	21	15	15
FSC threshold	0.143	0.143	0.143
Map resolution range (Å)	10-30	10-20	10-20
