# Supplementary material for: In situ structure and assembly of the multidrug efflux pump AcrAB-TolC
Source: Nat Commun. 2019 Jun 14;10:2635. doi: 10.1038/s41467-019-10512-6 (PMC6570770; doi:10.1038/s41467-019-10512-6)
Supplement: Supplementary file 3 — Description of Additional Supplementary Files [file 41467_2019_10512_MOESM3_ESM.docx]

**Title: Supplementary Movie 1
Description:** Side view and top view of the AcrAB-TolC efflux pump in the E. coli cell envelope. Slice view through a typical Cryo-ET of the E. coli overexpressing the AcrAB-TolC multidrug efflux pump, showing top and side views of the particles.
